# Establishment of oral microbiome in very low birth weight infants during the first weeks of life and the impact of oral diet implementation

**DOI:** 10.1371/journal.pone.0295962

**Published:** 2023-12-15

**Authors:** Pedro A. R. Vanzele, Luiz Gustavo Sparvoli, Patricia P. de Camargo, Carla R. Tragante, Glenda P. N. S. Beozzo, Vera L. J. Krebs, Ramon V. Cortez, Carla R. Taddei

**Affiliations:** 1 Department of Clinical and Toxicological Analyses, School of Pharmaceutical Sciences, University of São Paulo, São Paulo, SP, Brazil; 2 Neonatal Intensive Care Center, Children’s Institute, Hospital das Clínicas, São Paulo Medical School, University of São Paulo, São Paulo, SP, Brazil; 3 School of Arts, Sciences and Humanity, University of São Paulo, São Paulo, SP, Brazil; 4 Division of Clinical Laboratory, University Hospital ‐ University of São Paulo, São Paulo, SP, Brazil; UAE University: United Arab Emirates University, UNITED ARAB EMIRATES

## Abstract

Very low birth weight (VLBW) infants, mostly preterm, have many barriers to feeding directly from the mother’s breast, and need to be fed alternatively. Feeding is a major influencer in oral microbial colonization, and this colonization in early life is crucial for the promotion of human health. Therefore, this research aimed to observe the establishment of oral microbiome in VLBW infants during their first month of life through hospitalization, and to verify the impact caused by the implementation of oral diet on the colonization of these newborns. We included 23 newborns followed during hospitalization and analyzed saliva samples collected weekly, using *16S rRNA* gene sequencing. We observed a significant decrease in richness and diversity and an increase in dominance over time (q-value < 0.05). The oral microbiome is highly dynamic during the first weeks of life, and beta diversity suggests a microbial succession in early life. The introduction of oral diet does not change the community structure, but affects the abundance, especially of *Streptococcus*. Our results indicate that although time is related to significant changes in the oral microbial profile, oral feeding benefits genera that will remain colonizers throughout the host’s life.

## Introduction

Low birth weight (LBW, <2500 g) is one of the main causes of neonatal mortality and morbidity in low- and middle-income countries, along with preterm birth, infections, and asphyxia at birth [1]. This term also includes very low birth weight (VLBW, <1500 g) infants, and it is described that the lower the birth weight and gestational age, the greater the chances of death in early life [2].

Most VLBW infants are premature, and enteral feeding is often a challenge, due to their immature physiological system and neurological development [3, 4]. Thus, these newborns need to be initially fed by alternative routes, such as gastric tubes, which lead the milk directly to the intestine. Therefore, there is no direct contact of the milk with oral cavity, until the baby reaches maturity for implementation of oral diet [5].

The oral microbiome colonization begins from species able to adhere to epithelial cells, such as *Streptococcus salivarius* [6], and these will serve as an adhesion site for subsequent colonizers, through co-aggregation and co-adhesion mechanisms [7]. The presence of some microorganisms can create a niche for the establishment of others [8], as seen with several *Streptococcus* species (*S*. *gordonii*, *S*. *mitis*, *S*. *oralis*, *S*. *sanguinis* [7], and *S*. *salivarius* [6]), which start the colonization in oral biofilm, to ensure an adequate pathway for this process in this crucial window of time. Furthermore, the oral microbiome is referred as a potential biomarker for health in a local and systemic manner [9], and once initial colonization can persist over time, the microbiome preservation early in life is essential for promoting human health. The occurrence of dysbiosis, mediated by the presence of *Porphyromonas gingivalis* or an increased abundance of *Fusobacterium nucleatum* [10], can induce or predispose certain diseases, such as periodontitis, which causes a deregulated inflammatory response that potentiates many damages. This process is already related to the emergence of autoimmune reactions [11], oral cancer [12], and systemic diseases [13].

In addition to all the challenges preterm newborns face, they are also more susceptible to being colonized by oral pathogens, such as methicillin-resistant *Staphylococcus aureus*, as described in the study by Underwood and Sohn [14], which compared full-term neonates to extremely low birth weight babies. This is because these babies are restricted to a colonization by microorganisms often found in Neonatal Intensive Care Units (NICUs), since they remain hospitalized in these NICUs for a considerable time and lack important factors that help their initial microbial colonization [15, 16], such as the contact with their parents [17] and exposure to a healthier and more diverse environment [18].

After birth, some variables can affect the oral microbiome in very low birth weight infants, for example, the delivery mode [19]. Also, preterm infants present an oral microbiome composition different from term infants in early life, but within three months their microbiome stabilizes and reflects a similar pattern [20]. Although some studies have already evaluated the oral microbiome of preterm and/or VLBW infants [21], we barely know how oral diet implementation can influence the colonization of the oral microbiome in these infants during the early life period.

Due to their prematurity, VLBW infants stay in NICUs for a long time, and need to be fed by alternative routes until oral feeding is possible. Therefore, the purpose of this study was to evaluate the establishment of oral microbiome in VLBW infants during their first month of life through hospitalization in a NICU, considering the role of oral diet implementation.

## Materials and methods

### Ethics statement

The study was approved by the Research Ethics Committee of the Hospital das Clínicas of the University of São Paulo (Sao Paulo, Brazil) (CAAE: 09673619.7.0000.0068) and the newborns’ parents were informed about the study during recruitment and signed a written informed consent form stating their agreement with the research.

### Study design and subjects

This is a longitudinal, observational study, which included VLBW infants hospitalized in the NICU of Hospital das Clínicas of the University of São Paulo (Sao Paulo, Brazil). We collected weekly samples from 23 preterm babies born with very low birth weight, who were hospitalized in a NICU for at least four weeks. All newborns included were delivered by cesarean section and received oral diet at some point during hospitalization, which consisted of expressed human milk or milk-based infant formula for preterm. The chances of death in early life increases as lower the birth weight and gestational age of the infant [2], what demonstrate the needed to study this group. Also, we only included infants delivered by cesarian section due to most of the preterm babies with very low birth weight are delivered through this method [22]. The samples collection was conducted between February 11th–August 6th, 2019.

### Sample collection and DNA extraction

All saliva samples were collected using two sterile swabs, rubbing them in the baby’s oral cavity for about 30 seconds, covering the cheeks and tongue. The first sample was collected within the first 24 hours after birth, and subsequent collections were carried out weekly. For this study, we selected samples collected in the first, third and fourth postpartum weeks, and in the weeks immediately before and after the implementation of oral diet. After collection, the cotton swabs were transferred to a 15-ml tube containing 500 μl of Phosphate-Buffered Saline (PBS, pH 7.4) and stored at -20°C until shipped, within 24 hours after collection, to the Molecular Microbiology Laboratory, where they were stored at -80°C until DNA extraction was performed.

Initially, the samples were pre-treated as described by Cortez et al. [23], which basically consisted of adding 500 μl of 0.1% Dithiothreitol (DTT) solution to the samples, vortexing for 1 minute and incubating for 10 minutes at room temperature. The suspension was then centrifuged, the supernatant was discarded, and the pellet was resuspended in 200 μl of TELS buffer (20mg/mL lysozyme, 1M Tris-HCl (pH 7.5), 0.5 M EDTA (pH 8.0), 20% sucrose). Then, DNA extraction was performed with the QIAamp DNA blood mini kit (Qiagen), according to the manufacturer’s protocol. The extracted DNA was eluted in 80 μl of autoclaved ultrapure type I water (Milli-Q®) and stored at -80°C until use. As a negative control, we used a sterile swab opened at the laboratory and kept in air for 30 seconds, then transferred to a 1.5-ml tube with 500 μl of PBS solution, and DNA extraction was performed using the same procedures described for samples. The amount of DNA was quantified using Qubit 4 Fluorometer (ThermoFisher).

### *16S rRNA* library preparation and sequencing

The *16S rRNA* region amplified by PCR technique was V3-V4, with the primers 5′- CCTACGGGNGGCWGCAG-3′ forward and 5′-GACTACHVGGGTATCTAATCC-3 reverse to the described region [24], with Illumina adapters. The amplicons were pooled and loaded onto Illumina MiSeq clamshell style cartridge kit V2 with 500 cycles, for paired-end 250 sequencing at a final concentration of 10 pM. The library was clustered to a density of approximately 820 k/mm^2^. The MiSeq platform was used for image analysis, base calling, and data quality assessment.

### Bioinformatic analysis

*16S rRNA* amplicons were analyzed using software QIIME2 version 2021.11 (Quantitative Insights Into Microbial Ecology) [25]. Chimeric artifacts removal, sequencing alignment, and sequence quality control were executed using DADA2 plugin [26], with both forward and reverse sequences truncated at 245 nucleotides. Taxonomic assignment of Amplicon Sequence Variants (ASVs) was performed using the q2-feature-classifier resource [27] and the Bayes naive classify-sklearn taxonomy classifier against SILVA database version 138, adopting a 99% similarity [28, 29]. Diversity analyses and community comparisons were performed using R packages *qiime2R* [30], *phyloseq* [31], *microbiome* [32], and *vegan* [33] in the R software (version 4.1.2). For relative abundance analysis and graphical representation, we selected the ten most abundant bacterial genera, and the less representative genera were grouped into “others”. Alpha diversity is a numeric value that summarize the structure of the community for a single sample, with respect to the number of different organisms (richness), their abundance (evenness), or both [34]. In this research, it was determined by the indices Chao1 for richness [35] (with values converted to log10 to provide a better graphical representation), Shannon for diversity [36], and Simpson for dominance [37]. Beta diversity analysis is a way to quantifies similarity or distance among microbiomes [38], and it was determined observing communities’ unique fractions through unweighted and weighted UniFrac metrics.

### Statistical analyses

Statistical analyses were performed using R software (version 4.1.2), by observing samples over time (first, third, and fourth postpartum weeks) and in relation to the introduction of oral feeding (before and after). Relative abundance of the representative genera and alpha diversity were analyzed by linear model regression using *MASS* [39] and *jtools* [40] R packages. To select which variable was included in the adjusted models, they were individually tested and those with p-value < 0.05 were selected, both for alpha (S1 Table) and beta diversity (S2 Table). The adjusted model of alpha diversity and weeks after birth included oral feeding (yes/no) as covariate. Regarding oral diet, sepsis (yes/no) and gestational antibiotic use (yes/no) were selected as covariates for alpha diversity analysis. False Discovery Rate (FDR) correction was applied, and we used a q-value ≤ 0.10 as a significant level. To compare beta diversity over time and by oral feeding, permutation test PERMANOVA (adonis2 test; *vegan* R package [33]) was performed, with adjustment for confounding variables oral feeding (yes/no), time without oral feeding (yes/no), and sepsis (yes/no). Principal Coordinate Analysis (PCoA) graphics were created to visualize interaction of the communities, and a p-value ≤ 0.05 was considered significant for beta diversity. The heatmap of the ASVs abundance was constructed with the R package *ComplexHeatmap* [41], and the ASVs were selected to be included in the plot based on the ASVs significantly associated with the postpartum weeks (q-value < 0.25) using generalized linear models (*Maaslin2* R package [42]) with a filter for the ASVs with a minimum prevalence of 25%. The shared ASVs diagram was constructed using the *VennDiagram* R package [43]. All graphical representations were performed using the *ggplot2* package in R [44].

## Results

The newborns were assigned to a single group for analysis, which consisted of observing the effect of time on microbial colonization, and how diet could affect the oral microbiome of these babies. For this purpose, a total of 23 VLBW infants were included, with samples collected in the first (n = 19), third (n = 21) and fourth (n = 18) postpartum weeks, and also in the week before (n = 22) and the week after (n = 23) the implementation of oral diet, totaling 89 samples. For some babies, the sample from weeks before and after the implementation of oral diet coincided with the third or fourth hospitalization week. The variation in the number of samples during follow-up is due to some loss during the experiment conduction, since some samples did not have sufficient DNA concentration or did not amplify in the PCR step.

### Clinical data

All newborns enrolled in this study were preterm, with a mean gestational age of 30.52 weeks (± 2.98; 24–35 weeks) (Table 1), and a mean weight of 1134.30 grams (± 257.46; 612–1500 grams) at birth. These babies had to stay hospitalized for about 7.27 weeks (± 3.38; 3.14–17.86 weeks) and waited around 4.70 weeks (± 3.17; 1–15 weeks) for the introduction of oral diet. Therefore, the sample collection interval from birth to before oral diet implementation was about 28.50 days (± 20.25; 7–94 days), and for samples collected between before and after oral diet introduction, it was around 14.90 days (± 11.01; 6–38 days). Of the total diet administered orally to these babies, only about 24.90% (± 24.12%; 0–76.32%) was mother’s breast milk, while the remainder was formula. The mothers of these babies had a mean age of 33.48 (± 5.16; 23–42 years), and only 21.74% of them used antibiotics during pregnancy. More than half of the newborns (69.57%) included in the study underwent antibiotic therapy, although the rate of sepsis occurrence was 43.48%. The other infants received antibiotics as a prophylaxis. Among the 16 babies who underwent antibiotic therapy, more than half (56.25%) received the combination of penicillin and aminoglycoside, and the vast majority of babies (62.50%) underwent only one antibiotic therapy cycle, administered mainly at the first week of life. The S3 Table shows all the data on antibiotic therapy used by the newborns.

**Table 1:** Descriptive data of newborns enrolled in the study (n = 23).

Variable	Mean ± Standard Deviation	Min-Max
Birth weight (grams)	1134.30 ± 257.46	612–1500
Gestational age (weeks)	30.52 ± 2.98	24–35
Hospitalization (weeks)	7.27 ± 3.38	3.14–17.86
Weeks without oral diet	4.70 ± 3.17	1–15
Sampling interval from birth to before diet (days)	28.50 ± 20.25	7–94
Sampling interval from before diet to after diet (days)	14.90 ± 11.01	6–38
Breast milk (%)	24.90 ± 24.12	0–76.32
Maternal age (years)	33.48 ± 5.16	23–42
	**N**	**%**
Gestational antibiotic use		
Yes	5	21.74%
No	18	78.26%
Antibiotic use		
Yes	16	69.57%
No	7	30.43%
Sepsis		
Yes	10	43.48%
No	13	56.52%

### Microbiome composition and relative abundance

**Fig 1:**
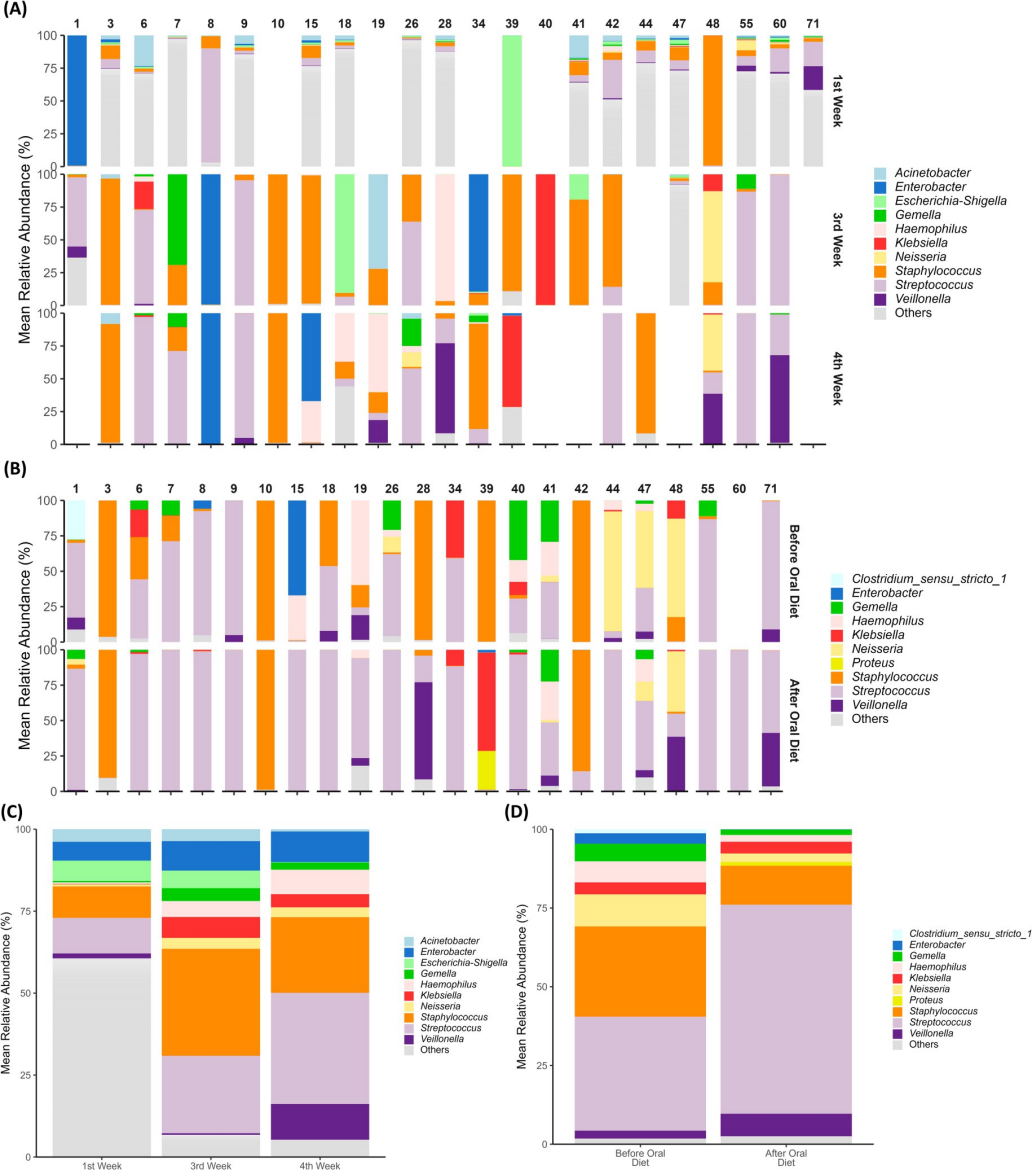
Mean relative abundance of the main genera observed in the samples by (A) postpartum week (first, third, and fourth weeks, as shown in the rows) and (B) oral diet (before and after introduction, as shown in the rows), depicted per infant (columns). (C) Stacked bar graphs showing the mean relative abundance of genera (%) in the infants’ oral microbiome in the first, third and fourth postpartum weeks. (D) Stacked bar graphs showing the mean relative abundance of genera (%) in the infants’ oral microbiome before and after the implementation of oral diet.

During the first four weeks of the baby’s life, the oral microbiome showed an adaptation over time (Fig 1A). There was a marked decrease in microbiome diversity within the first two weeks of life, and the individual’s microbiome community was dominated by one or two genera, most of them members of *Streptococcus*, *Staphylococcus* and Enterobacteriaceae members, by the third week of life. In the fourth week of life, when most babies had already started oral diet, the dominance of Enterobacteriaceae decreased and *Streptococcus* and *Staphylococcus* remained dominant (Fig 1A). The average relative abundance of genera corroborated individual data, demonstrating that the oral microbiome was mostly composed of less abundant genera, herein represented as “others”. In the first week of life, the greatest abundances were *Streptococcus* (10.86%), *Staphylococcus* (9.59%), *Escherichia-Shigella* (6.23%), *Enterobacter* (5.74%), and *Acinetobacter* (3.84%). Over time, there was an increase in the relative abundance of *Staphylococcus* (32.64%), *Streptococcus* (23.62%), *Enterobacter* (9.00%), *Klebsiella* (6.38%), *Haemophilus* (4.86%), *Gemella* (3.93%), and *Neisseria* (3.33%), and a slightly decrease in the abundance of *Escherichia-Shigella* (5.35%) and *Acinetobacter* (3.63%). In the fourth postpartum week, *Streptococcus* (33.90%) became the most abundant genus in babies’ oral microbiome, followed by *Staphylococcus* (23.07%), *Veillonella* (10.91%), *Enterobacter* (9.36%), *Haemophilus* (7.47%), *Klebsiella* (4.02%), *Neisseria* (3.00%), and *Gemella* (2.17%). The genera *Acinetobacter* (0.69%) and *Escherichia-Shigella* (0.13%) along with *Staphylococcus*, had a greater decrease when compared to the third postpartum week (Fig 1C). However, none of these data showed a statistically significant value related to relative abundance changes over time (S4 Table).

By assessing the oral microbiome of VLBW infants right after the implementation of oral diet, an increase in *Streptococcus* dominance (Fig 1B) was found in the analysis of the individual microbiome. The mean values of genera abundance before and after the implementation of oral diet were not statistically significant different; however, there was a marked increase in the abundance of *Streptococcus* (36.19% to 66.39%). Other genera showed a decrease in abundance after introduction of oral diet, as *Staphylococcus* (28.68% to 12.38%), *Neisseria* (10.18% to 2.69%), *Haemophilus* (6.70% to 2.16%), *Klebsiella* (3.79% to 3.73%), *Gemella* (5.58% to 1.70%), *Enterobacter* (3.33% to 0.09%), and *Clostridium_sensu_stricto_1* (1.26% to 0.01%), as shown in Fig 1D (S5 Table).

**Fig 2:**
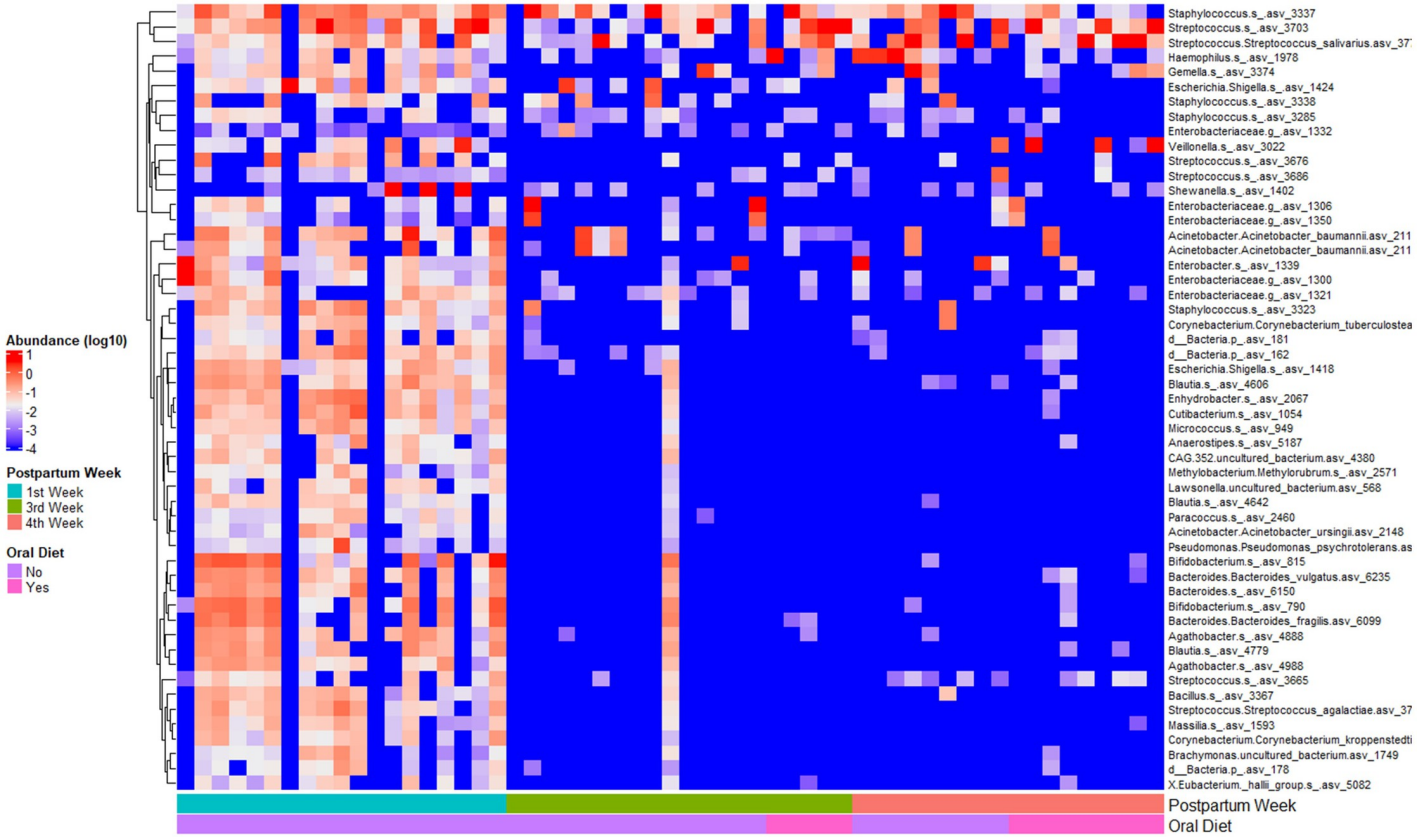
Heatmap of the Amplicon Sequencing Variants (ASVs) abundance (y-axis) significantly associated with the postpartum weeks (first, third and fourth week) by oral diet status (no/yes) (x-axis) using generalized linear models (Maaslin) and filtered by ASVs with a minimum prevalence of 25%.

When observed the taxa composition at Amplicon Sequencing Variants (ASV) level, we found ASVs that were significantly associated with the postpartum weeks. In addition to the genus abundance results, there is a decrease of the diversity over time, mainly from the first to the further weeks of life, with a slightly increase in the abundance of the ASVs of bacteria described as oral eobiont colonizers (Fig 2), but this is not associated with the oral diet implementation.

### Alpha diversity

**Fig 3:**
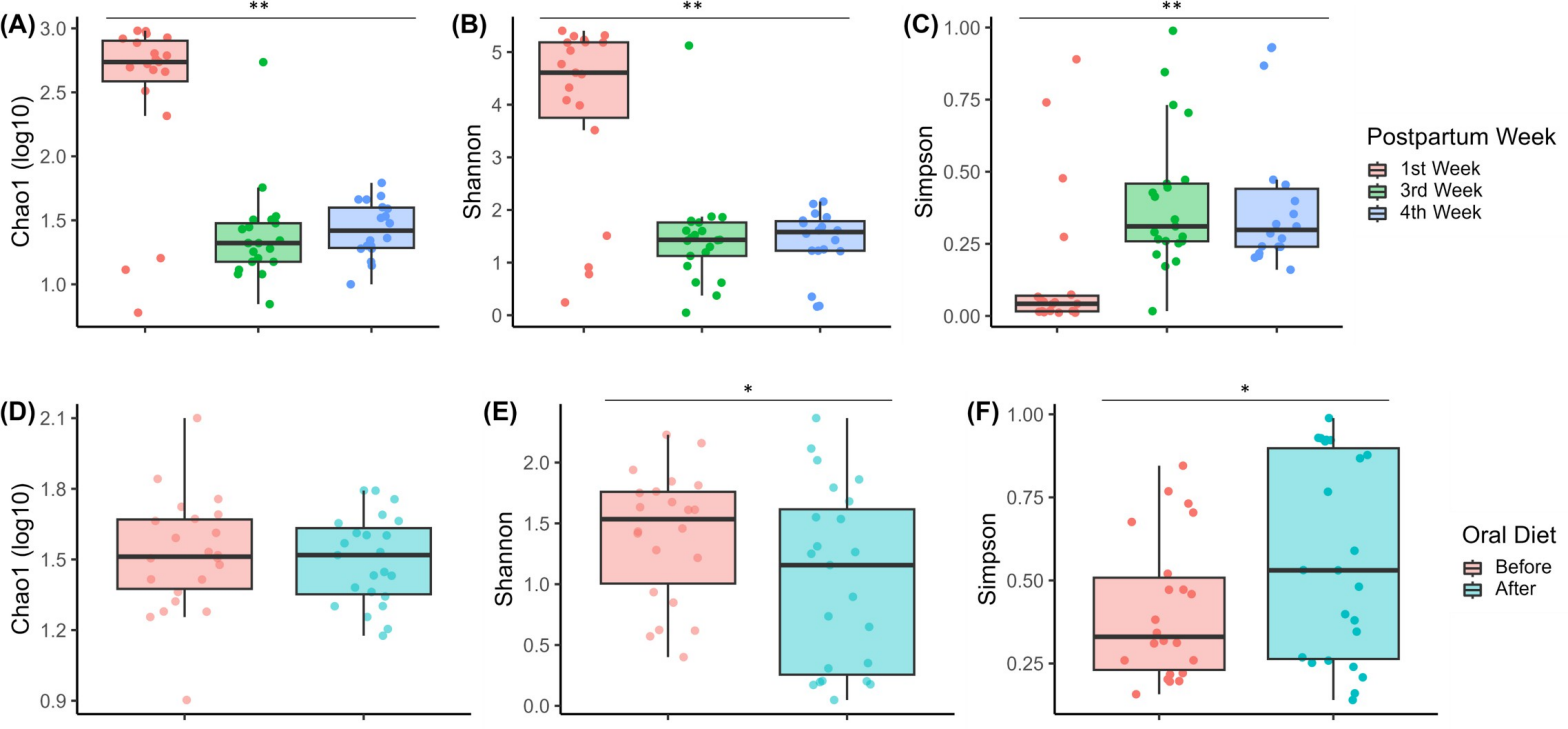
Boxplots showing the alpha diversity indices of Chao1(log10), Shannon, and Simpson, according to (A-C) postpartum weeks and (D-F) introduction of oral diet. Linear regression model testing was used to calculate p-value, and q-value results were confirmed with False Discovery Rate (FDR) *post-hoc*. The q-values were considered significant when ≤ 0.10. ^*^q-value < 0.10; ^**^q-value < 0.05.

The alpha diversity analysis, regarding postpartum weeks, showed that the oral microbiome is richer and more diverse in the first week, and that richness and diversity, as measured by Chao1 (2.50 ± 0.16 to 1.38 ± 0.08 to 1.43 ± 0.05; Fig 3A) and Shannon (3.96 ± 0.4 to 1.47 ± 0.21 to 1.39 ± 0.14; Fig 3B) indices, respectively, decrease over the weeks. Linear model testing showed that these decreases were significantly associated with weeks of life (β = -0.404; β = -0.856, respectively; q-value < 0.001 for both). Furthermore, there was an increase in dominance, as assessed by the Simpson index (0.15 ± 0.06 to 0.40 ± 0.05 to 0.39 ± 0.06; Fig 3C), confirmed as statistically significant using linear model testing (β = 0.062; q-value = 0.041). These results are shown in the S6 Table.

After oral feeding, the oral microbiome diversity is decreased (1.40 ± 0.11 to 1.04 ± 0.15; Fig 3E), in contrast with an increase in dominance (0.41 ± 0.05 to 0.56 ± 0.06; Fig 3F), both statistically significant (β = -0.362 and β = 0.150, respectively; q-value = 0.095 for both) (S7 Table). There was no difference in richness before and after the implementation of oral diet (1.52 ± 0.05 to 1.5 ± 0.04; Fig 3D), which means that there is no association of Chao1 index with oral diet (β = -0.022; q-value = 0.707).

### Beta diversity

**Fig 4:**
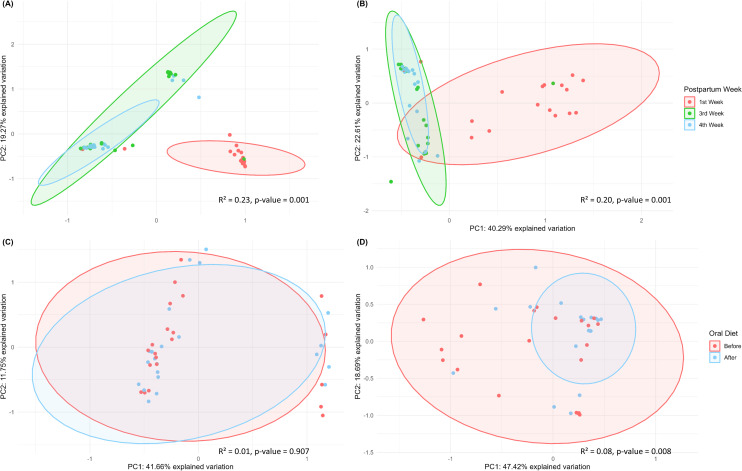
Principal coordinate analysis (PCoA) for (A) unweighted (R² = 0.23, p-value = 0.001) and (B) weighted (R² = 0.20, p-value = 0.001) Unifrac distance metrics over time (first, third and fourth week postpartum); (C) unweighted (R² = 0.01, p-value = 0.907) and (D) weighted (R² = 0.08, p-value = 0.008) Unifrac distance metrics, before and after implementation of oral diet.

Significant differences in beta diversity (based on unweighted Unifrac ‐ Fig 4A) were observed over time, with the babies’ first week samples clustered further apart from the others (R² = 0.23; p-value = 0.001). This was also found on beta diversity according to the weighted Unifrac metric, as shown in Fig 4B (R² = 0.20; p-value = 0.001).

Concerning the implementation of oral diet, no significant differences were found in the babies’ microbiome by unweighted Unifrac metric (R² = 0.01; p-value = 0.907) (Fig 4C). However, the weighted Unifrac demonstrates that there are statistical differences between the samples collected before and after oral diet (R² = 0.08; p-value = 0.008), with oral communities clustering closer after diet implementation than before (Fig 4D). Considering the impact of both postpartum weeks and oral diet status during the first weeks of life, we noticed that the time has greater effect in beta diversity than diet (S1 Fig).

### Shared ASVs

**Fig 5:**
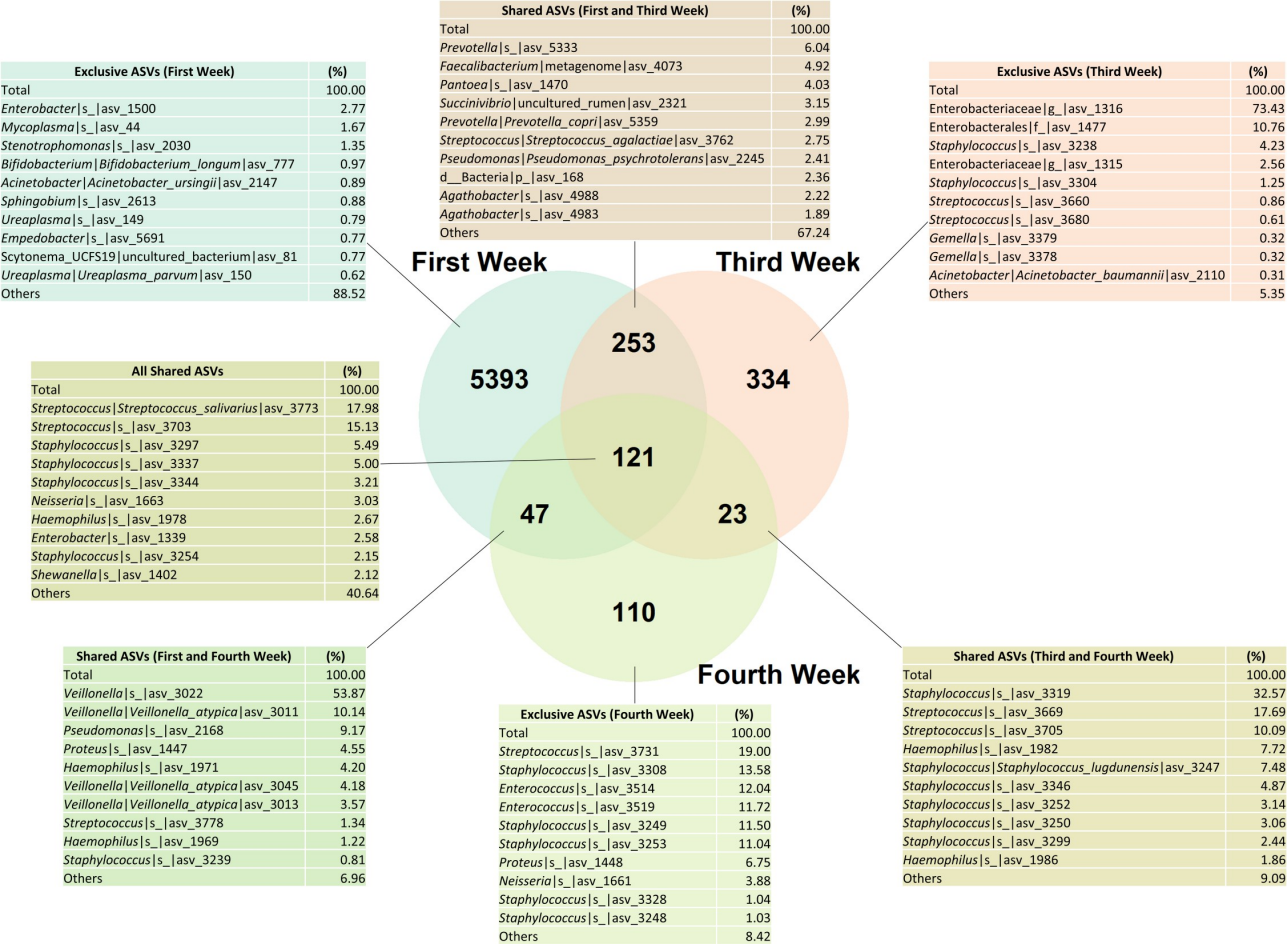
Shared and exclusive Amplicon Sequence Variants (ASVs) between the postpartum weeks (first, third, and fourth week).

The shared ASVs diagram (Fig 5) shows which ASVs were consistent in the oral microbiome during the first weeks of life. The microbiome composition of the first postpartum week has the greater richness and diversity, with 5393 exclusive ASVs at this point, and without a dominance of specific ASVs. In contrast, the third week has only 334 exclusive ASVs, with the main ASVs being Enterobacteriaceae members (73.43%). The fourth week has the lowest number of exclusive ASVs, but the greater abundance was of *Streptococcus*’ ASVs (19.00%). In addition, 121 ASVs were shared among all the first four weeks of life, and the most prevalent ASVs were of oral eobiont microorganisms, such as *Streptococcus*, *Staphylococcus*, *Neisseria* and *Haemophilus*. The main ASV shared in the first month (from all the weeks enrolled in this study) is from the *Streptococcus* genus, with ASVs of the *Streptococcus salivarius* species being the most prevalent among them.

Concerning the shared ASVs between the samples before and after the oral diet implementation, we observe that the 277 exclusive ASVs before the oral diet were consisted of Enterobacteriaceae members (71.21%), similar to the third postpartum week. After the oral diet, there is the introduction of 233 ASVs, mostly of oral eobiont colonizers such as *Veillonella* and *Streptococcus*. 164 ASVs were common both before and after the oral diet introduction, and it was also consisted of microorganism described as oral colonizers, mainly from the *Streptococcus* genus (S2 Fig).

## Discussion

This research evaluated the process of colonization of the oral microbiome in preterm VLBW infants in a NICU environment at different times, from birth to the implementation of oral diet, to observe the establishment of the oral microbiome. We emphasize here the decrease of genera associated with healthcare-associated infections through the weeks, and an increase in oral eobiont colonizers, especially after oral diet introduction. Also, there is a decrease in diversity and richness over time in contrast with an increase in dominance, according to alpha diversity indices, and the same was observed after oral diet implementation, except for richness. Structural changes in microbial communities were also observed, mainly driven by the decrease of abundance of some dominant genera, as seen in weighted Unifrac analysis, in both oral diet introduction and time variables.

In the delivery room, the newborn’s mouth is exposed to a diverse load of microorganisms, which are provided by breathing, and interaction with parents and hospital staff [6]. During the first weeks of life, we observed that the oral cavity is colonized by a highly diverse microbial community, especially in the first week, as evidenced by the high diversity in both relative abundance and alpha diversity data. The main genera observed in VLBW infants were *Streptococcus*, *Staphylococcus* and Enterobacteriaceae members, while *Acinetobacter*, *Haemophilus* and *Neisseria* showed lower relative abundance. In hospitalized premature newborns, microbes present in the hospital environment were also found as oral colonizers, such as *Escherichia*, *Staphylococcus*, and *Streptococcus* [45]. The authors also identified *Acinetobacter*, *Haemophilus*, and *Neisseria* in lower relative abundance colonizing the environment. In our study, we found similar bacteria in the babies’ mouth, suggesting that these genera may be acquired from an environmental source. However, to state this more categorically, a sampling of the environment would be necessary.

In the relative abundance transition between the first and the third weeks of life, there was a decrease in microbial diversity and richness, and this data is supported by the significant differences observed in Shannon and Chao1 indices over the weeks. This finding is associated to a microbiome shift related to the increase of oral eobiont genera, such as *Streptococcus*, *Veillonella*, and *Haemophilus*. In addition, there was a decrease in genera such as *Staphylococcus*, *Acinetobacter*, *Escherichia-Shigella*, and *Klebsiella*, described as colonizers of the hospital environment [46, 47]. This result suggests that there is a microbial succession occurring in the oral microbiome of hospitalized VLBW infants over time, regardless of oral feeding, also evidenced in beta diversity results. A previous publication by our group looking at the effect of oropharyngeal colostrum administration in premature newborns found similar results [23].

Newborns’ microbial communities evolve as the baby grows [48] and this dynamicity was observed in the relative abundance of genera during hospitalization. Despite the changes observed over time in the oral microbiome of early life, oral feeding also plays a role in establishing the microbiome. We found a decrease in the abundance of genera associated to health-related infections, such as *Staphylococcus*, *Klebsiella*, and *Enterobacter*, and an increase in the abundance of *Veillonella* and, mostly *Streptococcus*, which becomes the dominant genus when evaluating the genera relative abundance before and after the implementation of oral diet. The individual graph of genera relative abundance over time and in relation to diet status shows that oral microbiome in early life is widely dynamic and with greater inter-individual variations. However, the oral microbiome tends to acquire a certain stability over time, shifting to a more similar pattern between individuals after the implementation of oral diet.

Most of the oral diet given to the babies was preterm infant formula, in which maltodextrin is one of the most abundant carbohydrates. It is described that some species of the *Streptococcus* genus have genes encoding proteins associated with carbohydrate metabolism, specifically maltosaccharides [49]. For example, the gene *malP* encodes the enzyme maltodextrin phosphorylase [50], which performs a catalytic reaction of phosphorolysis of an alpha-1,4-glycosidic bond in maltodextrins [51]. Also, there is a putative maltodextrin-binding protein encoded by the *malE* gene described by the authors as an important factor for oropharyngeal colonization by *Streptococcus* [49].

Over the weeks, genera described as colonizers of the human oral cavity appear and stand out in relation to the others [48, 52], mostly *Streptococcus*. Cortez et al. [23], observed, in hospitalized newborns receiving oropharyngeal colostrum administration, higher levels of *Streptococcus* relative abundance around the 21^st^ day of life, slightly higher compared to the standard care group. Although we also observed an increased relative abundance of *Streptococcus*, this occurred around the 5^th^ week of life, suggesting that colostrum administration favors an early increase in the abundance of this genus.

Oral microbiome colonization begins with species able to adhere to epithelial cells and salivary proteins, such as *Streptococcus salivarius* [6], also known as the most abundant specie from the *Streptococcus* genus in newborns’ oral cavity [53]. We found that 121 ASV were shared in oral babies’ microbiome over time, and 168 ASV shared before and after oral diet introduction. Interestingly, most of them were *Streptococcus salivarius* in both analyzes. This specie will serve as an adhesion site for subsequent colonizers [7], which suggests that the presence of some microorganisms can create a niche for the establishment of others [8], thus providing an increased diversity and formation of more stable and complex communities. This genus has another important role in oral microbiome, since the commensal *Streptococcus* species can compete with pathogenic species for substrate, as *Porphyromonas gingivalis*, to prevent their adhesion and inhibit their growth [54, 55].

Since most babies in our study were not breastfed, we cannot state how human milk could impact the formation of the oral microbiome in these VLBW infants. However, we found a lack of important microorganisms, such as *Lactobacillus*, a genus associated with oral colonization in neonates fed exclusively or partially with human milk. This genus has properties that can inhibit the growth of pathogenic *Streptococcus* species, such as cariogenic *Streptococcus mutans* [56]. Additionally, human milk provides many elements for babies, due to its composition of macronutrients (proteins, lipids, and carbohydrates), micronutrients (vitamins), and bioactive components (cells, immunoglobulins, cytokines, chemokines, growth factors, hormones, and antimicrobial proteins) [57], besides its own source of probiotics (human milk microbiota) and prebiotics (human milk oligosaccharides (HMOs)) [58].

As limitations of our study, we can point out that the small sample size, along with the loss of some follow-up samples, impacted the statistical results. The sequence methodology used (16s rRNA gene sequencing) also is a limitation, as it does not allow us to go beyond the genus level for many microorganisms. In addition, the low number of babies fed with human milk did not allow us to attribute differences in the microbial profile to the intake of human milk. Also, all included infants were caesarean born, therefore our results cannot be generalized to the entire population, especially vaginally born VLBW infants. However, as strengths of our study, we can point the follow-up of the infants in different time points and the sampling before and after the oral diet implementation, which allowed us to compare how this type of diet could impact in the microbiome establishment in the oral cavity.

All these findings highlight the importance of stimulating oral feeding as early as possible in VLBW infants, mainly through an oropharyngeal colostrum administration protocol, which is very well described for its safety, simplicity and potential beneficial effects, with no additional risk to infants [59]. Many studies [48, 52, 60] shows that the oral microbiome fluctuates in early life along with the introduction of new colonizing factors, like solid food or tooth eruption. We could not specify how our findings can influence the short- and long-term health, but it is not possible to exclude that a colonization at this moment of life can determine a worse prognosis for the infants. Therefore, as perspectives, studies comparing the oral microbiome of VLBW preterm infants and full-term infants over the first year of life are needed to bring new and stronger hypotheses about how it could affect the establishment of the oral microbiome.

## Conclusions

In conclusion, our results suggest that, although time is related to significant changes in the oral microbial profile of very low birth weight babies, the implementation of oral diet also plays a major role in the initial establishment of microbiome, favoring specific genera that will remain as oral eobiont colonizers throughout the host’s life, as shown with *Streptococcus* relative abundance.

## Supporting information

S1 Fig Principal coordinate analysis (PCoA) for (A) unweighted (Postpartum Week: R² = 0.23, p-value = 0.001; Oral Diet: R² = 0.01, p-value = 0.923;) and (B) weighted (Postpartum Week: R² = 0.20, p-value = 0.001; Oral Diet: R² = 0.01, p-value = 0.365;) Unifrac distance metrics over time (first, third and fourth week postpartum) and according to the oral diet status (before and after).
(TIF)

S2 Fig Shared and exclusive Amplicon Sequence Variants (ASVs) before and after the oral diet implementation. (TIF)

S1 Table Covariates tested to be included in the adjusted model for alpha diversity analysis (p-value < 0.05 were selected). (DOCX)

S2 Table Covariates tested to be included in the adjusted model for beta diversity analysis (p-value < 0.05 were selected). (DOCX)

S3 Table Data on the use of antibiotic therapy by the study subjects. (DOCX)

S4 Table Distribution of the main observed bacterial genera according to postpartum weeks. ^#^p-value was based on linear model test, and q-value results were confirmed with False Discovery Rate (FDR) *post-hoc*.
(DOCX)

S5 Table Distribution of the main observed bacterial genera according to oral diet introduction. ^#^p-value was based on linear model test, and q-value results were confirmed with False Discovery Rate (FDR) *post-hoc*.
(DOCX)

S6 Table Alpha diversity metrics considering the postpartum weeks. ^#^p-value was based on a linear model test, and q-value results were confirmed with False Discovery Rate (FDR) *post-hoc*. ^*^ q-value was considered significant when ≤ 0.10.
(DOCX)

S7 Table Alpha diversity indices considering oral diet (before and after implementation). ^#^p-value was based on a linear model test, and q-value results were confirmed with False Discovery Rate (FDR) *post-hoc*. ^*^q-value was considered significant when ≤ 0.10.
(DOCX)
