# Supplementary figures and images for: Establishment of oral microbiome in very low birth weight infants during the first weeks of life and the impact of oral diet implementation
Source: PLoS One. 2023 Dec 15;18(12):e0295962. doi: 10.1371/journal.pone.0295962 (PMC10723731; doi:10.1371/journal.pone.0295962)

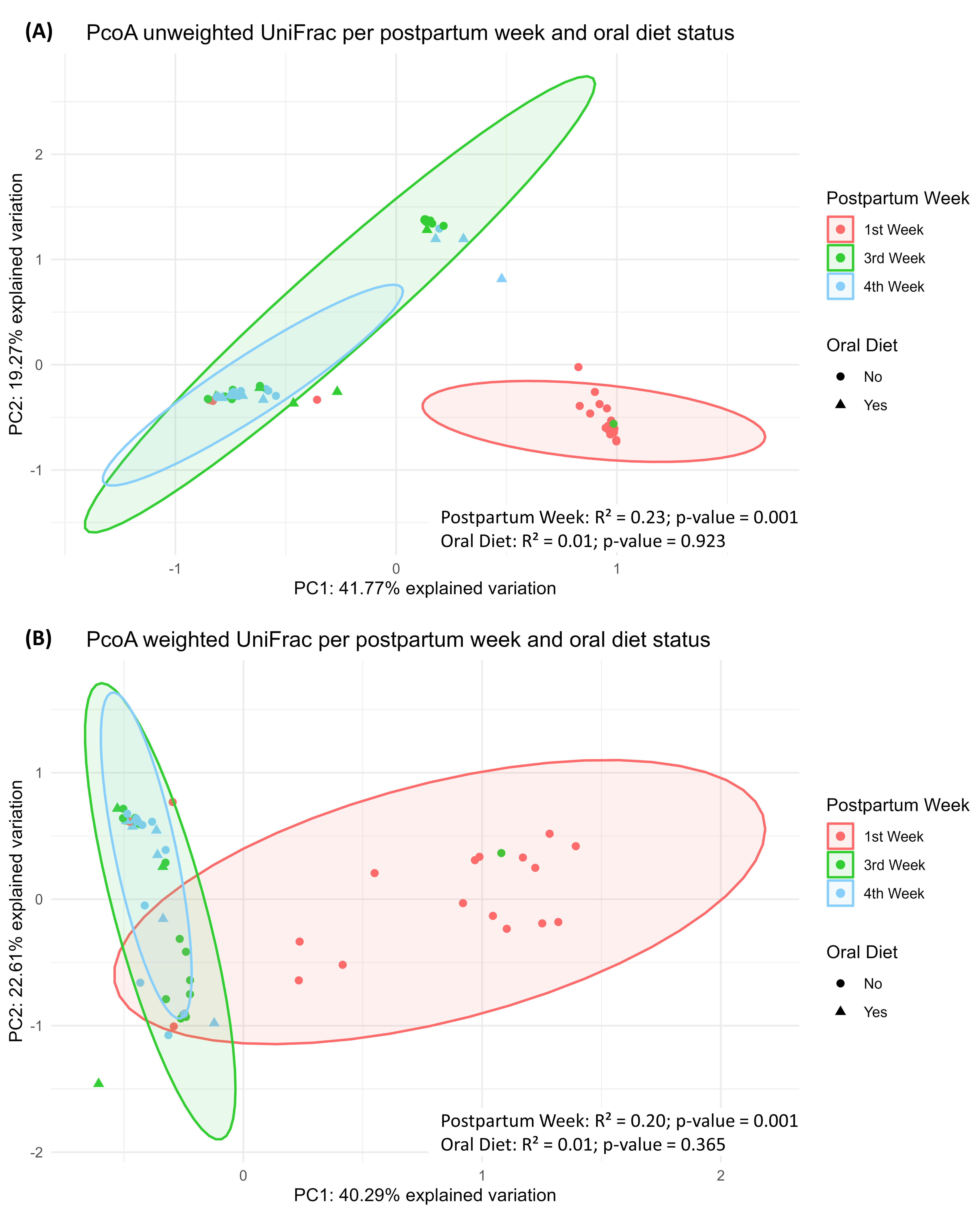

Supplement: S1 Fig — Principal coordinate analysis (PCoA) for (A) unweighted (Postpartum Week: R² = 0.23, p-value = 0.001; Oral Diet: R² = 0.01, p-value = 0.923;) and (B) weighted (Postpartum Week: R² = 0.20, p-value = 0.001; Oral Diet: R² = 0.01, p-value = 0.365;) Unifrac distance metrics over time (first, third and fourth week postpartum) and according to the oral diet status (before and after). (TIF) [file pone.0295962.s001.tif]

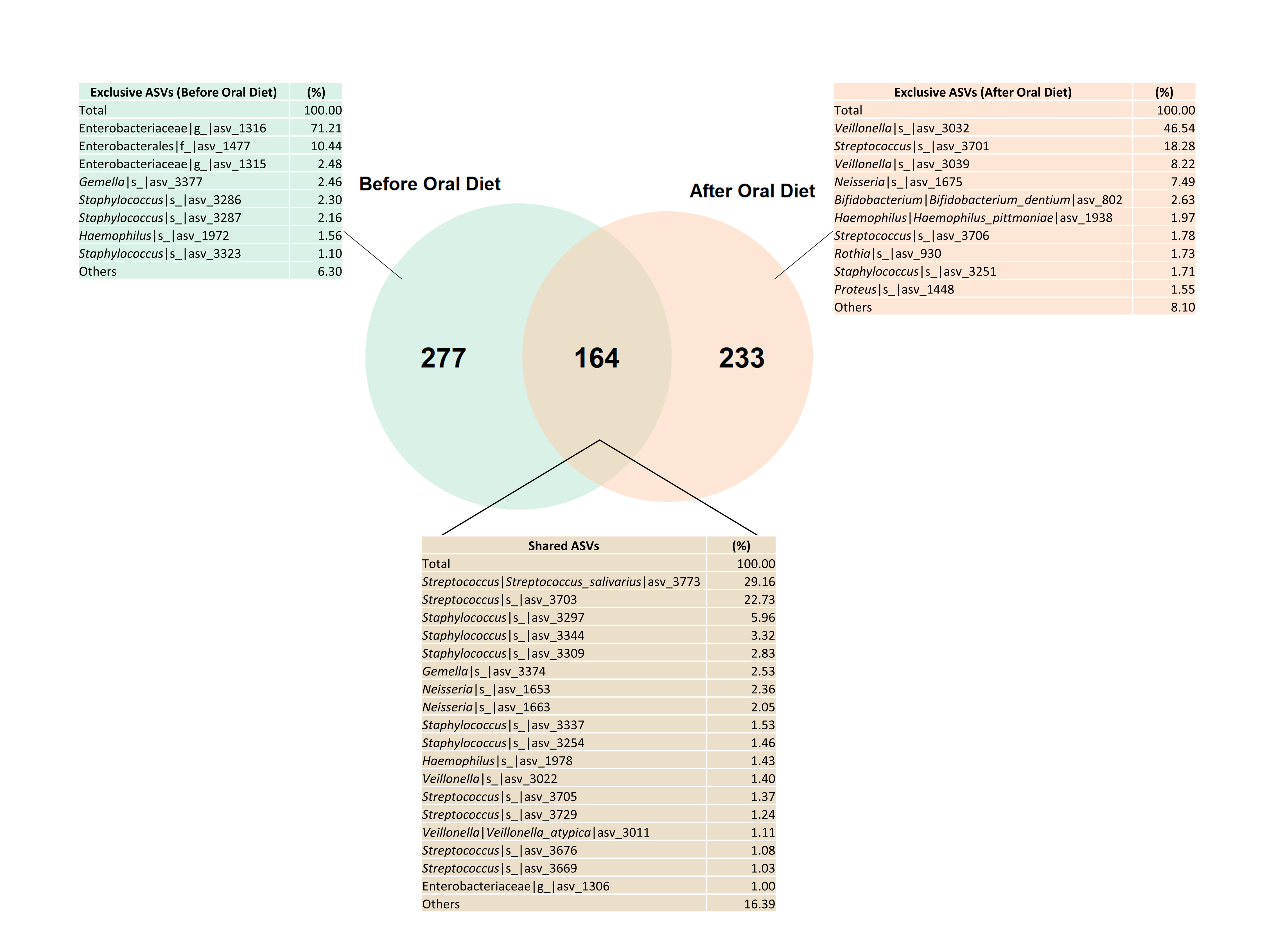

Supplement: S2 Fig — (TIF) [file pone.0295962.s002.tif]
