# Supplementary material for: Establishment of oral microbiome in very low birth weight infants during the first weeks of life and the impact of oral diet implementation
Source: PLoS One. 2023 Dec 15;18(12):e0295962. doi: 10.1371/journal.pone.0295962 (PMC10723731; doi:10.1371/journal.pone.0295962)
Supplement: S1 Table — (DOCX) [file pone.0295962.s003.docx]

**S1 Table.** Covariates tested to be included in the adjusted model for alpha diversity analysis (p-value < 0.05 were selected).

| **Variable** | **Indices** | **Covariate** | **p-value** |
| --- | --- | --- | --- |
| **Time** | **Chao1** | Breast milk intake | 0.506 |
|  |  | Sepsis | 0.784 |
|  |  | **Oral diet** | **0.040** |
|  |  | Gestational antibiotic use | 0.839 |
|  |  | Antibiotic use | 0.555 |
|  |  | Antibiotic association | 0.882 |
|  |  | Time without oral diet | 0.851 |
|  | **Shannon** | Breast milk intake | 0.311 |
|  |  | Sepsis | 0.437 |
|  |  | **Oral diet** | **0.003** |
|  |  | Gestational antibiotic use | 0.922 |
|  |  | Antibiotic use | 0.214 |
|  |  | Antibiotic association | 0.670 |
|  |  | Time without oral diet | 0.458 |
|  | **Simpson** | Breast milk intake | 0.769 |
|  |  | Sepsis | 0.455 |
|  |  | **Oral diet** | **0.003** |
|  |  | Gestational antibiotic use | 0.624 |
|  |  | Antibiotic use | 0.136 |
|  |  | Antibiotic association | 0.691 |
|  |  | Time without oral diet | 0.051 |
| **Diet** | **Chao1** | Breast milk intake | 0.427 |
|  |  | **Sepsis** | **0.017** |
|  |  | Time (postpartum week) | 0.554 |
|  |  | **Gestational antibiotic use** | **0.010** |
|  |  | Antibiotic use | 0.972 |
|  |  | Antibiotic association | 0.480 |
|  |  | Time without oral diet | 0.518 |
|  | **Shannon** | Breast milk intake | 0.773 |
|  |  | Sepsis | 0.157 |
|  |  | Time (postpartum week) | 0.884 |
|  |  | Gestational antibiotic use | 0.264 |
|  |  | Antibiotic use | 0.980 |
|  |  | Antibiotic association | 0.807 |
|  |  | Time without oral diet | 0.913 |
|  | **Simpson** | Breast milk intake | 0.952 |
|  |  | Sepsis | 0.174 |
|  |  | Time (postpartum week) | 0.789 |
|  |  | Gestational antibiotic use | 0.414 |
|  |  | Antibiotic use | 0.821 |
|  |  | Antibiotic association | 0.627 |
|  |  | Time without oral diet | 0.905 |
