# Supplementary material for: Establishment of oral microbiome in very low birth weight infants during the first weeks of life and the impact of oral diet implementation
Source: PLoS One. 2023 Dec 15;18(12):e0295962. doi: 10.1371/journal.pone.0295962 (PMC10723731; doi:10.1371/journal.pone.0295962)
Supplement: S2 Table — (DOCX) [file pone.0295962.s004.docx]

**S2 Table.** Covariates tested to be included in the adjusted model for beta diversity analysis (p-value < 0.05 were selected).

| **Variable** | **Metrics** | **Covariate** | **p-value** |
| --- | --- | --- | --- |
| **Time** | **Unweighted Unifrac** | **Oral Diet (Yes or No)** | **0.021** |
|  |  | Sepsis | 0.648 |
|  |  | Breast milk intake | 0.694 |
|  |  | Gestational antibiotic use | 0.447 |
|  |  | Antibiotic use | 0.587 |
|  |  | Antibiotic association | 0.717 |
|  |  | Time without oral diet | 0.333 |
|  | **Weighted Unifrac** | **Oral Diet (Yes or No)** | **0.003** |
|  |  | Sepsis | 0.095 |
|  |  | Breast milk intake | 0.218 |
|  |  | Gestational antibiotic use | 0.499 |
|  |  | Antibiotic use | 0.266 |
|  |  | Antibiotic association | 0.451 |
|  |  | Time without oral diet | 0.162 |
| **Diet** | **Unweighted Unifrac** | Time (postpartum weeks) | 0.584 |
|  |  | Sepsis | 0.202 |
|  |  | Breast milk intake | 0.185 |
|  |  | Gestational antibiotic use | 0.220 |
|  |  | Antibiotic use | 0.636 |
|  |  | Antibiotic association | 0.138 |
|  |  | **Time without oral diet** | **0.039** |
|  | **Weighted Unifrac** | Time (postpartum weeks) | 0.619 |
|  |  | **Sepsis** | **0.029** |
|  |  | Breast milk intake | 0.833 |
|  |  | Gestational antibiotic use | 0.321 |
|  |  | Antibiotic use | 0.383 |
|  |  | Antibiotic association | 0.175 |
|  |  | Time without oral diet | 0.088 |
