# Supplementary material for: Establishment of oral microbiome in very low birth weight infants during the first weeks of life and the impact of oral diet implementation
Source: PLoS One. 2023 Dec 15;18(12):e0295962. doi: 10.1371/journal.pone.0295962 (PMC10723731; doi:10.1371/journal.pone.0295962)
Supplement: S3 Table — (DOCX) [file pone.0295962.s005.docx]

**S3 Table.** Data on the use of antibiotic therapy by the study subjects.

| Variables | | Individuals (n = 16) | (%) |
| --- | --- | --- | --- |
| Antibiotic association | Penicillin and Aminoglycoside | 9 | 56.25 |
|  | Aminoglycoside and Glycopeptide | 3 | 18.75 |
|  | Penicillin and Cephalosporin | 2 | 12.50 |
|  | Aminoglycoside and Glycopeptide / Cephalosporin | 1 | 6.25 |
|  | Penicillin and Aminoglycoside / Glycopeptide | 1 | 6.25 |
| Antibiotic therapy cycle | 1 cycle | 10 | 62.50 |
|  | 2 cycles | 4 | 25.00 |
|  | 3 cycles | 1 | 6.25 |
|  | 4 cycles | 1 | 6.25 |
