# Supplementary material for: Establishment of oral microbiome in very low birth weight infants during the first weeks of life and the impact of oral diet implementation
Source: PLoS One. 2023 Dec 15;18(12):e0295962. doi: 10.1371/journal.pone.0295962 (PMC10723731; doi:10.1371/journal.pone.0295962)
Supplement: S4 Table — #p-value was based on linear model test, and q-value results were confirmed with False Discovery Rate (FDR) post-hoc. (DOCX) [file pone.0295962.s006.docx]

**S4 Table.** Distribution of the main observed bacterial genera according to postpartum weeks.

| Genera | | Relative Abundance (%) | | |
| --- | --- | --- | --- | --- |
|  |  | **1st Week (n = 19)** | **3rd Week (n = 21)** | **4th Week (n = 18)** |
| *Streptococcus* | Min-Max | 0 - 86.90 | 0 - 99.75 | 0 - 99.96 |
|  | Mean | 10.87 | 23.62 | 33.91 |
|  | Estimate | 5.032 | | |
|  | 95% CI | -2.947 – 13.011 | | |
|  | q-value^#^ | 0.534 | | |
| *Staphylococcus* | Min-Max | 0.10 - 98.97 | 0.18 - 98.78 | 0.02 - 98.83 |
|  | Mean | 9.59 | 32.64 | 23.07 |
|  | Estimate | 5.933 | | |
|  | 95% CI | -2.444 – 14.310 | | |
|  | q-value^#^ | 0.534 | | |
| *Enterobacter* | Min-Max | 0 - 99.28 | 0 - 99.31 | 0 - 99.57 |
|  | Mean | 5.74 | 9.00 | 9.36 |
|  | Estimate | 3.696 | | |
|  | 95% CI | -2.497 – 9.889 | | |
|  | q-value^#^ | 0.534 | | |
| *Haemophilus* | Min-Max | 0 - 3.93 | 0 - 96.49 | 0 - 59.73 |
|  | Mean | 0.45 | 4.86 | 7.47 |
|  | Estimate | 2.136 | | |
|  | 95% CI | -1.680 – 5.953 | | |
|  | q-value^#^ | 0.534 | | |
| *Veillonella* | Min-Max | 0 - 18.07 | 0 - 8.44 | 0 - 68.66 |
|  | Mean | 1.49 | 0.48 | 10.92 |
|  | Estimate | 0.961 | | |
|  | 95% CI | -2.135 – 4.057 | | |
|  | q-value^#^ | 0.648 | | |
| *Escherichia-Shigella* | Min-Max | 0 - 99.78 | 0 - 90.44 | 0 - 1.86 |
|  | Mean | 6.23 | 5.35 | 0.13 |
|  | Estimate | -1.175 | | |
|  | 95% CI | -5.440 – 3.091 | | |
|  | q-value^#^ | 0.648 | | |
| *Klebsiella* | Min-Max | 0 - 0.66 | 0 - 99.59 | 0 - 69.47 |
|  | Mean | 0.15 | 6.38 | 4.02 |
|  | Estimate | 1.211 | | |
|  | 95% CI | -2.680 – 5.102 | | |
|  | q-value^#^ | 0.648 | | |
| *Acinetobacter* | Min-Max | 0 - 23.37 | 0 - 71.94 | 0 - 8.21 |
|  | Mean | 3.84 | 3.64 | 0.69 |
|  | Estimate | -0.548 | | |
|  | 95% CI | -2.995 – 1.900 | | |
|  | q-value^#^ | 0.656 | | |
| *Neisseria* | Min-Max | 0 - 7.73 | 0 - 69.48 | 0 - 42.75 |
|  | Mean | 0.64 | 3.33 | 3.00 |
|  | Estimate | 0.924 | | |
|  | 95% CI | -1.688 – 3.535 | | |
|  | q-value^#^ | 0.648 | | |
| *Gemella* | Min-Max | 0 - 1.49 | 0 - 69.12 | 0 - 20.82 |
|  | Mean | 0.39 | 3.93 | 2.17 |
|  | Estimate | 1.499 | | |
|  | 95% CI | -0.794 – 3.792 | | |
|  | q-value^#^ | 0.534 | | |

^#^p-value was based on linear model test, and q-value results were confirmed with False Discovery Rate (FDR) *post-hoc*.
