# Supplementary material for: Establishment of oral microbiome in very low birth weight infants during the first weeks of life and the impact of oral diet implementation
Source: PLoS One. 2023 Dec 15;18(12):e0295962. doi: 10.1371/journal.pone.0295962 (PMC10723731; doi:10.1371/journal.pone.0295962)
Supplement: S5 Table — #p-value was based on linear model test, and q-value results were confirmed with False Discovery Rate (FDR) post-hoc. (DOCX) [file pone.0295962.s007.docx]

**S5 Table.** Distribution of the main observed bacterial genera according to oral diet introduction.

| Genera | | Relative Abundance (%) | |
| --- | --- | --- | --- |
|  |  | **Before Oral Diet (n = 22)** | **After Oral Diet (n = 23)** |
| *Streptococcus* | Min-Max | 0.05 - 94.85 | 0.05 - 99.89 |
|  | Mean | 36.19 | 66.39 |
|  | Estimate | 21.402 | |
|  | 95% CI | -0.229 – 43.033 | |
|  | q-value^#^ | 0.262 | |
| *Staphylococcus* | Min-Max | 0.05 - 99.87 | 0.01 - 98.83 |
|  | Mean | 28.68 | 12.38 |
|  | Estimate | -6.889 | |
|  | 95% CI | -27.406 – 13.629 | |
|  | q-value^#^ | 0.558 | |
| *Veillonella* | Min-Max | 0 - 17.40 | 0 - 68.66 |
|  | Mean | 2.52 | 7.16 |
|  | Estimate | 5.195 | |
|  | 95% CI | -2.878 – 13.268 | |
|  | q-value^#^ | 0.407 | |
| *Neisseria* | Min-Max | 0 - 84.54 | 0 - 42.75 |
|  | Mean | 10.18 | 2.69 |
|  | Estimate | -7.188 | |
|  | 95% CI | -18.903 – 4.527 | |
|  | q-value^#^ | 0.407 | |
| *Haemophilus* | Min-Max | 0 - 59.73 | 0 - 27.20 |
|  | Mean | 6.69 | 2.16 |
|  | Estimate | -5.579 | |
|  | 95% CI | -12.600 – 1.442 | |
|  | q-value^#^ | 0.387 | |
| *Klebsiella* | Min-Max | 0 - 40.41 | 0 - 69.47 |
|  | Mean | 3.79 | 3.73 |
|  | Estimate | 0.651 | |
|  | 95% CI | -7.169 – 8.472 | |
|  | q-value^#^ | 0.867 | |
| *Gemella* | Min-Max | 0 - 42.03 | 0 - 22.42 |
|  | Mean | 5.58 | 1.70 |
|  | Estimate | -5.411 | |
|  | 95% CI | -10.606 – -0.215 | |
|  | q-value^#^ | 0.262 | |
| *Enterobacter* | Min-Max | 0 - 67.03 | 0 - 1.83 |
|  | Mean | 3.33 | 0.09 |
|  | Estimate | -3.185 | |
|  | 95% CI | -9.527 – 3.157 | |
|  | q-value^#^ | 0.453 | |
| *Clostridium_sensu_stricto_1* | Min-Max | 0 - 27.63 | 0 - 0.07 |
|  | Mean | 1.26 | 0.00 |
|  | Estimate | -1.017 | |
|  | 95% CI | -3.619 – 1.585 | |
|  | q-value^#^ | 0.544 | |
| *Proteus* | Min-Max | 0 - 0.10 | 0 - 27.19 |
|  | Mean | 0.00 | 1.18 |
|  | Estimate | 1.495 | |
|  | 95% CI | -1.057 – 4.048 | |
|  | q-value^#^ | 0.407 | |

^#^p-value was based on linear model test, and q-value results were confirmed with False Discovery Rate (FDR) *post-hoc*.
