# Supplementary material for: Establishment of oral microbiome in very low birth weight infants during the first weeks of life and the impact of oral diet implementation
Source: PLoS One. 2023 Dec 15;18(12):e0295962. doi: 10.1371/journal.pone.0295962 (PMC10723731; doi:10.1371/journal.pone.0295962)
Supplement: S6 Table — #p-value was based on a linear model test, and q-value results were confirmed with False Discovery Rate (FDR) post-hoc. * q-value was considered significant when ≤ 0.10. (DOCX) [file pone.0295962.s008.docx]

**S6 Table.** Alpha diversity metrics considering the postpartum weeks.

| Indices | | Alpha Diversity | | |
| --- | --- | --- | --- | --- |
|  |  | **1st Week (n = 19)** | **3rd Week (n = 21)** | **4th Week (n = 18)** |
| Chao1 (log10) | Mean ± Standard Error | 2.50 ± 0.16 | 1.38 ± 0.08 | 1.43 ± 0.05 |
|  | Estimate | -0.404 | | |
|  | 95% CI | -0.525 – -0.284 | | |
|  | q-value^#^ | 0.000^*^ | | |
| Shannon | Mean ± Standard Error | 3.96 ± 0.4 | 1.47 ± 0.21 | 1.39 ± 0.14 |
|  | Estimate | -0.856 | | |
|  | 95% CI | -1.157 – -0.555 | | |
|  | q-value^#^ | 0.000^*^ | | |
| Simpson | Mean ± Standard Error | 0.15 ± 0.06 | 0.40 ± 0.05 | 0.39 ± 0.06 |
|  | Estimate | 0.062 | | |
|  | 95% CI | 0.002 – 0.121 | | |
|  | q-value^#^ | 0.041^*^ | | |

^#^p-value was based on a linear model test, and q-value results were confirmed with False Discovery Rate (FDR) *post-hoc*. ^*^ q-value was considered significant when ≤ 0.10.
