# Supplementary material for: Establishment of oral microbiome in very low birth weight infants during the first weeks of life and the impact of oral diet implementation
Source: PLoS One. 2023 Dec 15;18(12):e0295962. doi: 10.1371/journal.pone.0295962 (PMC10723731; doi:10.1371/journal.pone.0295962)
Supplement: S7 Table — #p-value was based on a linear model test, and q-value results were confirmed with False Discovery Rate (FDR) post-hoc. *q-value was considered significant when ≤ 0.10. (DOCX) [file pone.0295962.s009.docx]

**S7 Table.** Alpha diversity indices considering oral diet (before and after implementation).

| Indices | | Alpha Diversity | |
| --- | --- | --- | --- |
|  |  | **Before Oral Diet (n = 22)** | **After Oral Diet (n = 23)** |
| Chao1 (log10) | Mean ± Standard Error | 1.52 ± 0.05 | 1.50 ± 0.04 |
|  | Estimate | -0.022 | |
|  | 95% CI | -0.138 – 0.094 | |
|  | q-value^#^ | 0.707 | |
| Shannon | Mean ± Standard Error | 1.40 ± 0.11 | 1.04 ± 0.15 |
|  | Estimate | -0.362 | |
|  | 95% CI | -0.745 – 0.021 | |
|  | q-value^#^ | 0.095^*^ | |
| Simpson | Mean ± Standard Error | 0.41 ± 0.05 | 0.56 ± 0.06 |
|  | Estimate | 0.150 | |
|  | 95% CI | -0.008 – 0.307 | |
|  | q-value^#^ | 0.095^*^ | |

^#^p-value was based on a linear model test, and q-value results were confirmed with False Discovery Rate (FDR) *post-hoc*. ^*^q-value was considered significant when ≤ 0.10.
